# Association of *NOTCH3* Variant Risk Category With 2-Year Clinical and Radiologic Small Vessel Disease Progression in Patients With CADASIL

**DOI:** 10.1212/WNL.0000000000209310

**Published:** 2024-05-07

**Authors:** Minne N. Cerfontaine, Remco J. Hack, Benno Gesierich, Marco Duering, Marie-Noëlle W. Witjes-Ané, Mar Rodríguez-Girondo, Gido Gravesteijn, Julie Rutten, Saskia A.J. Lesnik Oberstein

**Affiliations:** From the Departments of Clinical Genetics (M.N.C., R.J.H., G.G., J.R., S.A.J.L.O.), Geriatrics and Psychiatrics (M.-N.W.W.-A.), and Medical Statistics (M.R.-G.), Leiden University Medical Center, the Netherlands; and Medical Image Analysis Center (MIAC) and Department of Biomedical Engineering (B.G., M.D.), University of Basel, Switzerland.

## Abstract

**Background and Objectives:**

Pathogenic variants in *NOTCH3* are the main cause of hereditary cerebral small vessel disease (SVD). SVD-associated *NOTCH3* variants have recently been categorized into high risk (HR), moderate risk (MR), or low risk (LR) for developing early-onset severe SVD. The most severe NOTCH3-associated SVD phenotype is also known as cerebral autosomal dominant arteriopathy with subcortical infarcts and leukoencephalopathy (CADASIL). We aimed to investigate whether *NOTCH3* variant risk category is associated with 2-year progression rate of SVD clinical and neuroimaging outcomes in CADASIL.

**Methods:**

A single-center prospective 2-year follow-up study was performed of patients with CADASIL. Clinical outcomes were incident stroke, disability (modified Rankin Scale), and executive function (Trail Making Test B given A *t*-scores). Neuroimaging outcomes were mean skeletonized mean diffusivity (MSMD), normalized white matter hyperintensity volume (nWMHv), normalized lacune volume (nLV), and brain parenchymal fraction (BPF). Cox regression and mixed-effect models, adjusted for age, sex, and cardiovascular risk factors, were used to study 2-year changes in outcomes and differences in disease progression between patients with HR-*NOTCH3* and MR-*NOTCH3* variants.

**Results:**

One hundred sixty-two patients with HR (n = 90), MR (n = 67), and LR (n = 5) *NOTCH3* variants were included. For the entire cohort, there was 2-year mean progression for MSMD (β = 0.20, 95% CI 0.17–0.23, *p* = 7.0 × 10^−24^), nLV (β = 0.13, 95% CI 0.080–0.19, *p* = 2.1 × 10^−6^), nWMHv (β = 0.092, 95% CI 0.075–0.11, *p* = 8.8 × 10^−20^), and BPF (β = −0.22, 95% CI −0.26 to −0.19, *p* = 3.2 × 10^−22^), as well as an increase in disability (*p* = 0.002) and decline of executive function (β = −0.15, 95% CI −0.30 to −3.4 × 10^−5^, *p* = 0.05). The HR-NOTCH3 group had a higher probability of 2-year incident stroke (hazard ratio 4.3, 95% CI 1.4–13.5, *p* = 0.011), and a higher increase in MSMD (β = 0.074, 95% CI 0.013–0.14, *p* = 0.017) and nLV (β = 0.14, 95% CI 0.034–0.24, *p* = 0.0089) than the MR-NOTCH3 group. Subgroup analyses showed significant 2-year progression of MSMD in young (n = 17, β = 0.014, 95% CI 0.0093–0.019, *p* = 1.4 × 10^−5^) and premanifest (n = 24, β = 0.012, 95% CI 0.0082–0.016, *p* = 1.1 × 10^−6^) individuals.

**Discussion:**

In a trial-sensitive time span of 2 years, we found that patients with HR-*NOTCH3* variants have a significantly faster progression of major clinical and neuroimaging outcomes, compared with patients with MR-*NOTCH3* variants. This has important implications for clinical trial design and disease prediction and monitoring in the clinic. Moreover, we show that MSMD is a promising outcome measure for trials enrolling premanifest individuals.

## Introduction

Small vessel disease (SVD) is an important cause of stroke and vascular dementia.^1^ Proaggregatory *NOTCH3* variants are a major genetic risk factor of SVD.^2-5^ These variants are present in minimally 1:300 individuals worldwide^6^ and are associated with an extremely broad disease spectrum, ranging from stroke before age 30 years to stroke and dementia-free survival beyond 80 years.^2,3,5,7-9^ The most severe end of the *NOTCH3*-associated SVD (NOTCH3-SVD) spectrum is also known as cerebral autosomal dominant arteriopathy with subcortical infarcts and leukoencephalopathy (CADASIL), which has a minimal prevalence of 2–5:100,000 individuals worldwide.^10^ In several cross-sectional studies, the position of the *NOTCH3* variant along the NOTCH3 protein's 34 epidermal growth factor-like repeat (EGFr) domains has been shown to be strongly associated with NOTCH3-SVD severity, in addition to classical cardiovascular risk factors (CVRF) and sex.^11-18^ Variants in NOTCH3 EGFr domains can be stratified into 3 risk categories, namely low risk (LR), moderate risk (MR), and high risk (HR) for developing severe NOTCH3-SVD.^13^ High-risk *NOTCH3* variants (HR-NOTCH3) are predominantly found in patients with CADASIL, whereas low-risk *NOTCH3* variants (LR-NOTCH3) are most frequent in healthy-volunteer population databases, such as UK Biobank. Moderate-risk *NOTCH3* variants (MR-NOTCH3) are prevalent in both CADASIL and population cohorts.^13^

Proaggregatory NOTCH3-SVD variants are almost exclusively cysteine-altering missense variants predicted to disrupt intra-EGFr disulfide bridge formation.^19^ This ultimately leads to NOTCH3 aggregation in the extracellular matrix of the (cerebral) microvasculature.^20^ Although the molecular mechanisms underlying the association between *NOTCH3* variant risk category and disease severity have not yet been elucidated, it has been shown that patients with CADASIL with HR-*NOTCH3* variants have higher levels of NOTCH3 aggregation in skin and brain vessels than patients with MR and LR-*NOTCH3* variants.^13,21^ This strongly supports the theory that NOTCH3 aggregation is the pathomechanistic driver of NOTCH3-SVD,^22^ setting into motion a cascade of events leading to destruction of cerebral small vessel wall integrity, and thereby reduced cerebrovascular reactivity^23^ and cerebral perfusion.^24^

Clinical manifestations of severe NOTCH3-SVD, or CADASIL, are mid-adult onset of ischemic stroke and transient ischemic attacks, migraine with aura, mood disorders, and cognitive decline, ultimately leading to vascular dementia.^25^ Neuroimaging features are progressive symmetrical white matter hyperintensities (WMHs), lacunes, cerebral microbleeds, perivascular spaces, and atrophy. White matter hyperintensities can precede clinical manifestations by decades.^26^ Diffusion tensor imaging (DTI) has been shown to sensitively capture white matter tract alterations, even in areas with normal-appearing white matter on T2-weighted imaging,^27^ suggesting that DTI may provide readily quantifiable biomarkers in early CADASIL/NOTCH3-SVD stages.

Cross-sectional studies have provided compelling evidence that *NOTCH3* variant risk category is a major modifier of NOTCH3-SVD severity,^11-18^ although this was not found in 2 studies of French patients.^28,29^ Prospective CADASIL follow-up studies taking into account NOTCH3 risk category have not been performed so far. It is, therefore, unknown whether NOTCH3 risk category is a predictor of disease progression. Determining to what extent NOTCH3 risk category predicts (short-term) disease progression is important to improve individualized disease prediction, to tailor disease monitoring and management, and to enable patient stratification in future clinical trials. We performed a single-center, prospective 2-year follow-up study to investigate whether NOTCH3 risk category is associated with clinical and neuroimaging disease progression in patients with CADASIL.

## Methods

### Study Participants

**Figure 1:**
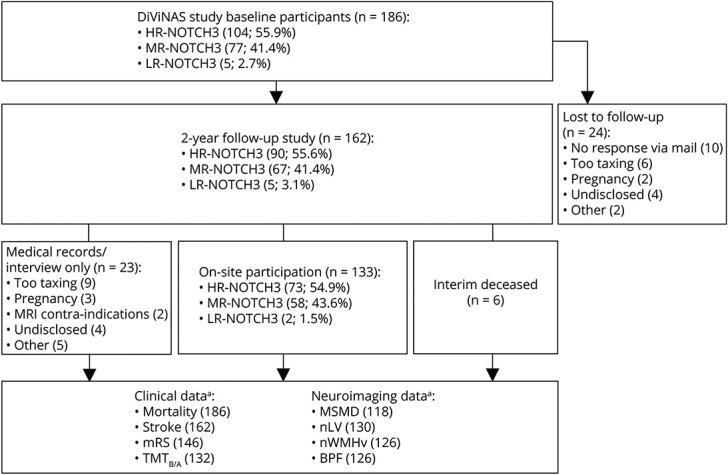
Flowchart of Inclusion and Loss to Follow-Up in the DiViNAS Study ^a^ See eMethods for detailed reasons of loss to follow-up. BPF = brain parenchymal fraction; DiViNAS = Disease Variability in *NOTCH3*-Associated Small Vessel Disease; HR-NOTCH3 = high-risk *NOTCH3* variant; MR-NOTCH3 = moderate-risk *NOTCH3* variant; mRS = modified Rankin Scale; MSMD = mean skeletonized mean diffusivity; nLV = normalized lacune volume; nWMHv = normalized white matter hyperintensity volume; TMT_B/A_ = Trail Making Test B given A *t*-scores.

Participants of the Disease Variability in *NOTCH3*-Associated Small Vessel Disease (DiViNAS) study were recruited from the Dutch CADASIL registry, which consists of patients and presymptomatic or paucisymptomatic family members with a genetically confirmed cysteine-altering *NOTCH3* variant. All patients were 20 years or older. Participants visited the LUMC at baseline^11^ and at 2-year follow-up between May 2019 and December 2023. Details concerning inclusion are presented in Figure 1. At both time points, participants were characterized on a single day, with cerebral MRI, neuropsychological testing, medical history, skin biopsy, and blood withdrawal. If patients were unable to participate in the on-site follow-up study, they were asked whether they were willing to provide 2-year follow-up information through a telephone interview and/or their medical records. This article follows the Strengthening the Reporting of Observational studies in Epidemiology (STROBE) guidelines.^30^

### Standard Protocol Approvals, Registrations, and Patient Consents

The DiViNAS study was approved by the Medical Ethics Committee Leiden-The Hague-Delft (P18.164 and P21.013). All participants gave written informed consent. All procedures were performed in accordance with ethical rules and the principles of the Declaration of Helsinki.

### NOTCH3 Risk Category Stratification

Individuals were stratified into NOTCH3 risk categories as previously described.^13^ HR-*NOTCH3* variants are located in EGFr domains 1–6, 8, 11 and 26; MR-*NOTCH3* variants in EGFr domains 9, 10, 12–15, 17, 25, 27, and 32; and LR-*NOTCH3* variants in EGFr domains 16, 18, 19, 20, 23, 24, 28–31, and 33. *NOTCH3* variant annotation and risk classification of participants of the DiViNAS study is presented in the eTable 1. Because there were only 5 participants with an LR-*NOTCH3* variant, these were excluded from comparative analyses.

### Mortality, Clinical, and Neuropsychological Measures

To determine whether patients were deceased during the follow-up period, the Dutch Personal Records Database was queried. For all patients, clinical information concerning cause of death and the disease course before death was available. Causes of death and premorbid disease course were further detailed by requesting medical records and by interviews with relatives.

Participants were assessed for a history of hypertension, hypercholesterolemia, diabetes type 1 or 2, and smoking status (see Table 1 and eMethods for details). Clinical outcomes were defined as previously described.^11^ Briefly, incident stroke during the follow-up period was assessed using medical history and was defined as either neurologic deficits that lasted longer than 24 hours in the absence of other probable causes or as a diagnosis of ischemic stroke in the medical records; disability was assessed using the modified Rankin Scale (mRS) questionnaire. A neuropsychological test battery was performed at both time points and included the Trail Making Test (TMT) Parts A and B. Scores of the TMT B were corrected for age, sex, educational level, and TMT A-score (TMT Part B given A *t*-scores, TMT_B/A_) using normative data reported in the literature.^31^ Patients who were unable to finish the TMT Part A or B in time (<300 seconds) because of too severe cognitive deficits (n = 7) were scored as the lowest *t*-score in the cohort, which was equal to 15. One patient was unable to complete the TMT because of impaired vision. mRS was available for 146 individuals and TMT_B/A_ for 132 at both time points (Figure 1).

**Table 1:** Baseline Descriptives of DiViNAS 2-Year Follow-Up Study Participants

	Total	HR-NOTCH3	MR-NOTCH3
No. of participants, n (%)	157 (100)	90 (57.3)	67 (42.7)
Follow-up time, mo, median (IQR)	24.7 (2.6)	25.4 (3.1)	24.3 (1.7)
Age at baseline, y, mean (SD)	51.1 (12.2)	48.9 (12.2)	54.1 (11.7)
Sex, male, n (%)	78 (49.7)	46 (48.9)	34 (50.7)
Education level (according to Verhage), n (%)			
Basic (0–4)	36 (22.9)	16 (17.8)	20 (29.9)
Average (5)	62 (39.5)	34 (37.8)	28 (41.8)
High (6–7)	59 (37.6)	40 (44.4)	19 (28.3)
Hypertension, n (%)	36 (22.9)	16 (17.8)	20 (29.9)
Hypercholesterolemia, n (%)	53 (33.8)	28 (31.1)	25 (37.3)
Diabetes type 1 or 2, n (%)	9 (5.7)	2 (2.2)	7 (10.4)
Current smoking, n (%)	16 (10.2)	9 (10)	7 (10.4)
No. of patients with 0, 1, 2 or 3 CVRF, n (%)			
0	75 (47.8)	48 (53.3)	27 (40.3)
1	57 (36.3)	31 (34.5)	26 (38.9)
2	18 (11.5)	9 (10)	9 (13.4)
3	7 (4.4)	2 (2.2)	5 (7.4)

Abbreviations: CVRF = cardiovascular risk factors; DiViNAS = Disease Variability in *NOTCH3*-Associated Small Vessel Disease; IQR = interquartile range.

### Neuroimaging Outcomes

Brain MRI scans at both time points were performed on the same 3T MR scanner (Philips Achieva TX, Philips Medical Systems, Best, the Netherlands) as described previously.^11^ Briefly, the following sequences were included in the protocol: 3D-T1-weighted (T1w) images, 3D-T2-weighted images, T2 fluid-attenuated inversion recovery (FLAIR), susceptibility-weighted images (SWIs), and DTI (eMethods).

SVD neuroimaging markers were quantified at both time points (eMethods). Briefly, for longitudinal image registration, a template was created per patient using T1w images of both time points as input. T1w images were rigidly registered to this template, and FLAIR, SWI, and TRACE (i.e., mean of diffusion-weighted images) were affine registered to the T1w images of the same time point in the template space. Longitudinally registered images were subtracted for each modality to calculate difference maps across time points for visual rating. Incident lacunes were scored according to the STRIVE criteria^32^ and were segmented to calculate changes in total lacune volume (LV) over time. Lacunes and LV at baseline were assessed as previously described.^11^

WMHs were segmented using a fully automated segmentation approach using FLAIR and registered 3D-Tw images as input. Intracranial and brain parenchymal volumes were determined from longitudinally registered T1w and FLAIR images. LV, WMH volume (WMHv), and brain volume were normalized to intracranial volume ([WMHv, LV and brain volume/intracranial volume] × 100) to calculate normalized WMHv (nWMHv), normalized LV (nLV), and brain parenchymal fraction (BPF).

Mean skeletonized mean diffusivity (MSMD) was chosen a priori as a marker for microstructural white matter damage (eMethods), as peak width of the skeletonized mean diffusivity^27^ (PSMD) is prone to software updates and thus not as robust in longitudinal analysis as MSMD on Philips MRI scanners.^33^ MSMD was calculated using a publicly available script and software container (eMethods). An additional sensitivity analysis was performed for PSMD. Three participants were excluded from MRI scanning at one or both time points because of contraindications. For the longitudinal analysis of MRI markers using both time points, nLV was available for 130 participants, nWMHv and BPF for 126 participants, and MSMD for 118 participants (Figure 1). Incident lacunes, nWMHv, and BPF were analyzed at 2 independent sites (Medical Image Analysis Center and Leiden University Medical Center), and intersite pipeline replicability was determined (eMethods). The MRI investigators (M.D., B.G., R.H. and M.C.) were blinded to the *NOTCH3* variant risk category.

### Statistics

Variables with a normal distribution were described using mean ± SD; variables with a skewed distribution were described using median ± interquartile range (IQR). In accordance with the STROBE guidelines,^30^ inferential measures including *p*-values were not reported in the baseline characteristics table. To reduce the number of predictors, instead of defining each cardiovascular risk factor separately, a variable number of CVRF (CVRFn) was created, which was defined as the total number of CVRF at baseline for each individual. CVRF included were hypertension, hypercholesterolemia, diabetes type 1 or 2, and current smoking status (for definitions, see eMethods). To analyze 2-year differences in cumulative incident stroke probability between the HR-NOTCH3 and MR-NOTCH3 groups, multivariable Cox regression was performed adjusted for sex, baseline age, and baseline CVRFn. Analysis of recurrence rate of lifetime stroke was performed using an Andersen-Gill Cox repeated-measures model, adjusted for the number of previous strokes, CVRF, and sex.

Statistical testing of continuous and ordinal outcomes was performed using mixed-effects models (MMs) with a random intercept. For continuous outcomes, these were linear mixed models (LMMs), and for the ordinal outcome mRS, this was a cumulative link mixed model (CLMM). Participants who had only one available observation for a particular outcome (either at baseline or follow-up) were also included in the MMs. Missing data were assumed to be missing at random. To achieve normal distribution or homoscedasticity of residuals, the following transformations were performed: natural logarithmic transformation of MSMD, cube root transformation of nLV, and square root transformation of nWMHv. For the LMMs, continuous dependent variables were standardized by subtracting the mean and dividing the value by the standard deviation.

For hypothesis testing, likelihood ratio testing was performed comparing MMs with and without the relevant interaction terms with follow-up time as a continuous covariate. To test whether the observed 2-year changes were statistically significant, a first MM was created per outcome with follow-up time, and compared with a null model. To assess the effect of NOTCH3 risk category on 2-year changes in study outcomes, an MM was then created with NOTCH3 risk category and its interaction with follow-up time and the following covariates: sex, baseline age, and baseline CVRFn. To test whether CVRFn and sex also influenced progression, an MM was fitted with additional interaction terms between CVRFn and follow-up time as well as sex and follow-up time. In case of differential progression between the HR and MR-NOTCH3 groups (i.e., a significant interaction term between NOTCH3 risk category and follow-up time), post hoc *t*-tests were performed within the MMs to test whether progression was also significant within each NOTCH3 risk category group.

As previous studies have shown that DTI may detect microstructural alterations in normal-appearing white matter^27^ and DTI outcomes would require the smallest sample size for demonstrating a treatment effect in CADASIL,^34^ additional subgroup analysis of progression of MSMD was performed using a paired *t*-test of log-transformed MSMD in the following groups: (1) patients younger than 40 years and (2) in premanifest individuals, defined as only minimal deep white matter hyperintensities (Fazekas score <2) and no lacunes at baseline. Baseline descriptives of the analyzed subgroups are provided in eTable 2.

Standardized coefficients (β), odds ratios (ORs) (for LMM: β_time_ and β_time × NOTCH3_, and for CLMM: OR_time × NOTCH3_), and hazard ratios were reported with 95% CIs and *p*-values. In the LMMs, a sensitivity analysis that included pedigree as an additional random effect was performed. If β_time × NOTCH3_ was significant, sensitivity analyses were performed with models that included the following additional interaction terms: (1) between follow-up time and each cardiovascular risk factor as an independent predictor and (2) between follow-up time and age to examine whether statistical inferences of NOTCH3 risk category would remain significant. Additional correction for multiple testing of the effect of NOTCH3 risk category on all outcomes (n = 7) was performed using a Benjamini-Hochberg procedure, and *q*-values were reported in Table 2. Two-sided *p*-values and *q*-values below 0.05 were considered significant. Statistical analysis was performed using R version 4.2.1.

**Table 2:** Results at Baseline and 2-Year Follow-Up

Clinical outcomes	HR-NOTCH3		MR-NOTCH3						
	Baseline	Follow-up	Baseline	Follow-up					
Ischemic stroke					**Hazard ratio_NOTCH3_**	***p*_NOTCH3_** ^**a**^	***p*_Age_** ^**a**^	***p*_Sex_** ^**a**^	***p*_CVRFn_** ^**a**^
No. of patients with at least 1 ischemic stroke (%)	29 (32.2)	36 (40.0)	12 (17.9)	13 (19.4)					
No. of patients with incident ischemic stroke (%)	—	17 (18.9)	—	4 (6.0)	4.2 (vs MR-NOTCH3)	0.011^b^	0.016	0.055	0.69
					***p*_time_** ^**c**^	***p*_time × NOTCH3_** ^**d**^			
mRS, n (%)					0.002	0.054			
0	40 (48.8)	36 (43.9)	29 (47.5)	28 (45.9)					
1–2	38 (46.3)	35 (42.7)	25 (41.0)	24 (39.3)					
≥3	4 (4.9)	11 (13.4)	7 (11.5)	9 (14.8)					
TMT_B/A_, mean (SD)	46.8 (11.9)	44.4 (12.8)	47.2 (12.0)	44.9 (11.8)	0.05	0.87			

Neuroimaging outcomes	**HR-NOTCH3**		**MR-NOTCH3**						
	**Baseline**	**Follow-up**	**Baseline**	**Follow-up**					
					***p*_time_** ^**c**^	***p*_time×NOTCH3_** ^**d**^	***p*_HR-NOTCH3_** ^**c**^	***p*_MR-NOTCH3_** ^**c**^	
MSMD, median (IQR)	8.5 × 10^−4^ (1.1 × 10^−4^)	8.7 × 10^−4^ (1.2 × 10^−4^)	8.2 × 10^−4^ (8.8 × 10^−5^)	8.3 × 10^−4^ (9.9 × 10^−5^)	7.0 × 10^−24^	0.017^b^	2.5 × 10^−20^	5.4 × 10^−10^	
nLV,^e^ median (IQR)	0.0028 (0.025)	0.0060 (0.027)	0 (0.0046)	0 (0.0065)	2.1 × 10^−6^	0.0089^b^	1.8 × 10^−7^	0.15	
nWMHv,^e^ median (IQR)	3.5 (5.0)	4.0 (5.4)	1.3 (2.3)	1.5 (2.8)	8.8 × 10^−20^	0.22			
BPF,^e^ mean (SD)	74.2 (3.7)	73.1 (4.0)	71.7 (3.4)	70.8 (3.6)	3.2 × 10^−22^	0.39			

Abbreviations: BPF = brain parenchymal fraction; CVRFn = number of cardiovascular risk factors at baseline; IQR = interquartile range; mRS = modified Rankin Scale; MSMD = mean skeletonized mean diffusivity; nLV = normalized lacune volume; nWMHv = normalized white matter hyperintensity volume; TMT_B/A_ = Trail Making Test B given A *t*-scores.

a *p*-Value derived from multivariable Cox regression with NOTCH3 risk category, sex, baseline age, and baseline CVRFn as covariates.

b The effect of NOTCH3 risk category on stroke probability and progression of MSMD and nLV remained statistically significant after correction for multiple testing using the Benjamini-Hochberg procedure (*q*-value = 0.039 for all 3 outcomes).

c Overall *p*-value derived from univariable mixed-effect models with follow-up time as covariate; if there was a significant difference in progression between the HR and MR-NOTCH3 risk categories (^d^), a *p*-value for time per risk category was also reported (*p*_HR-NOTCH3_ and *p*_MR-NOTCH3_, derived from the linear mixed model below^d^). For statistical analyses, MSMD was log-transformed, nWMHv was square root–transformed, and nLV was cube root–transformed.

d *p*-Value derived from multivariable mixed-effect models after correction for sex, baseline age, and baseline CVRFn.

e nWMHv, nLV, and BPF are normalized and are expressed as a percentage of the intracranial volume (in milliliters). The median (IQR) non-normalized WMH volume in the HR-NOTCH3 group was 55.1 mL (81.3) at baseline and 62.5 mL (79.4) at follow-up; for the MR-NOTCH3 group, this was 18.1 mL (35.5) at baseline and 21.8 mL (40.4) at follow-up.

### Data Availability

Data supporting the findings presented in this article are available on reasonable request from the corresponding authors.

## Results

### Baseline Descriptives and Loss to Follow-Up

Follow-up data were obtained of 162 patients of the 186 baseline DiViNAS participants. This included either an on-site visit for the full 2-year follow-up research protocol (n = 133) or clinical data acquisition through clinical records and/or telephone interviews (n = 29). Twenty-four patients were lost to follow-up (Figure 1). Of the 162 follow-up participants, 90 had an HR-*NOTCH3* variant, 67 an MR-*NOTCH3* variant, and 5 an LR-*NOTCH3* variant (eTable 1).

The median follow-up duration was 24.7 months (IQR 2.6 months, Table 1). At baseline, patients with MR-*NOTCH3* variants were older, had lower educational levels, and had a higher burden of CVFR compared to patients with HR-*NOTCH3* variants (Table 1). HR and MR-NOTCH3 participants did not differ in any of the other baseline descriptives.

### Two-Year Mortality

Three patients with an HR-*NOTCH3* variant and 3 with an MR-*NOTCH3* variant died in the interim. Four of these patients died because of CADASIL-related causes, namely stroke or end-stage CADASIL (HR-NOTCH3 ages 55 and 73 years, MR-NOTCH3 ages 71 and 73 years). Another patient died at 60 years due to myelofibrosis and one at 59 years due to (likely) acute myocardial infarction.

### Two-Year Changes in Clinical Measures

**Figure 2:**
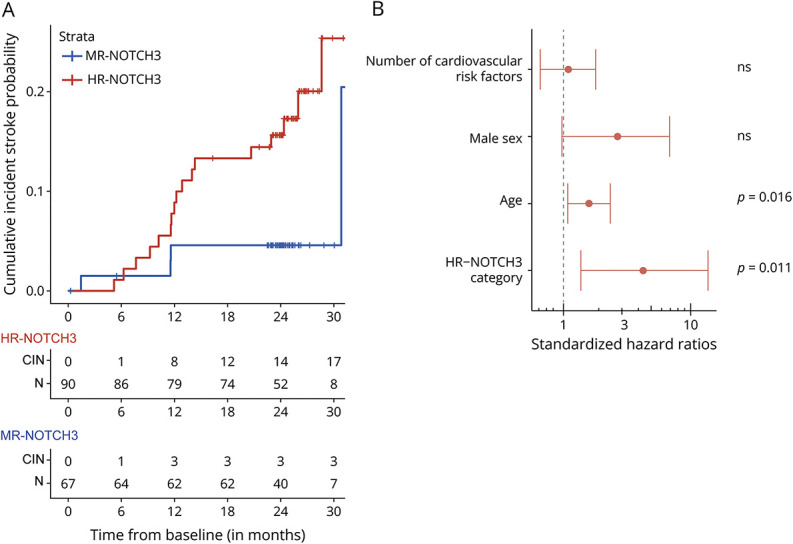
The HR-NOTCH3 Group Had a Higher 2-Year Cumulative Probability of Experiencing a Stroke Compared with the MR-NOTCH3 Group (A) Kaplan-Meier survival curve for incident stroke of HR-NOTCH3 (red) and MR-NOTCH3 patients (blue) with a table showing CIN of stroke and those at risk (N). One patient with an MR-*NOTCH3* variant who experienced stroke shortly after the cutoff of the CIN/N table; this patient was included in the Cox regression model. (B) Forest plot showing the relative effect sizes of the different predictors on 2-year incident cumulative stroke probability; only the HR-NOTCH3 group (hazard ratio HR-NOTCH3 vs MR-NOTCH3 4.3, 95% CI 1.4–13.5, *p* = 0.011) and age (standardized by 10 years, hazard ratio 1.6, 95% CI 1.1–2.4, *p* = 0.016) predicted 2-year incident stroke. CIN = cumulative incidence; HR-NOTCH3 = high-risk *NOTCH3* variant; MR-NOTCH3 = moderate-risk *NOTCH3* variant; N = number at risk.

In the 2-year follow-up period, 13.4% (n = 21) of participants had an incident ischemic stroke. In the HR-NOTCH3 group, this was 18.9% (n = 17), and in the MR-NOTCH3 group, this was 6.0% (n = 4). Two-year incident stroke probability was predicted by *NOTCH3* variant risk category (hazard ratio HR-NOTCH3 vs MR-NOTCH3: 4.3, 95% CI 1.4–13.5, *p* = 0.011) and age (hazard ratio 1.6, 95% CI 1.1–2.4, *p* = 0.016), but not by sex and CVRFn (Figure 2). Patients with HR-*NOTCH3* variants also had a higher lifetime risk of recurrent stroke (hazard ratio HR-NOTCH3 vs MR-NOTCH3: 3.0, 95% CI 1.7–5.4, *p* = 1.8 × 10^−4^) (eFigure 1).

There was an increase in mRS over time for the entire cohort (*p* = 0.002) (eFigure 2), with no difference between HR and MR-NOTCH3 patients (OR_time × NOTCH3_ HR-NOTCH3 vs MR-NOTCH3: 3.6, 95% CI 0.94–14.2, *p* = 0.054). For TMT_B/A_, there was a marginal change over time for the entire cohort (β_time_ = −0.15, 95% CI −0.30 to −3.4 × 10^−5^, *p* = 0.05). Changes in TMT_B/A_ were more pronounced in older patients (eFigure 2). There were no differences in progression rate of TMT_B/A_ between the 2 NOTCH3 risk category groups (β_time × NOTCH3_ = −0.024, 95% CI −0.33 to 0.28, *p* = 0.87). Sex and CVRFn did not significantly affect the 2-year change in TMT_B/A_ or mRS. Other CADASIL-related clinical signs and symptoms are summarized in eTable 3.

### Two-Year Changes in Neuroimaging Markers

**Figure 3:**
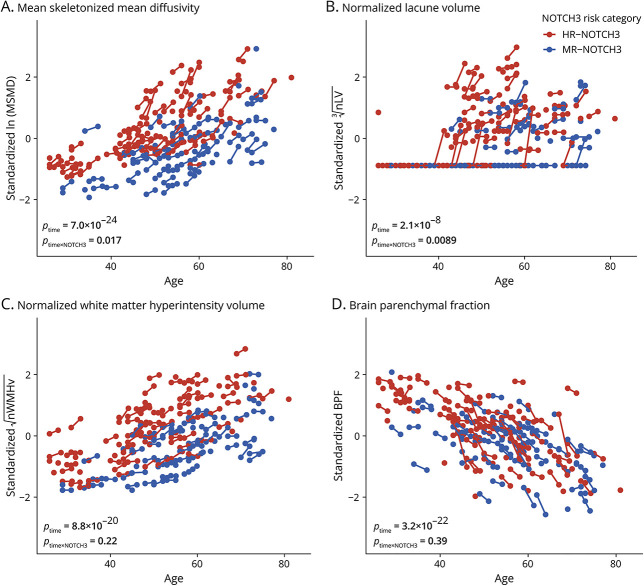
The HR-NOTCH3 Group Had Higher Mean 2-Year Progression of MSMD and nLV Compared With the MR-NOTCH3 Group (A–D) Spaghetti plots with standardized neuroimaging markers on the y-axis and age at study visit on the x-axis. There was an increase in MSMD (β_time_ = 0.20, 95% CI 0.17–0.23, *p* = 7.0 × 10^−24^, A), nLV (β_time_ = 0.13, 95% CI 0.080–0.19, *p* = 2.1 × 10^−6^, B), and nWMHv (β_time_ = 0.092, 95% CI 0.075–0.11, *p* = 8.8 × 10^−20^, C) and a decrease in BPF (β_time_ = −0.22, 95% CI −0.26 to −0.19, *p* = 3.2 × 10^−22^, D) for the entire cohort. Patients with HR-*NOTCH3* variants (in red) had a higher mean increase of MSMD (β_time × NOTCH3_ = 0.074, 95% CI 0.013–0.14, *p* = 0.017) and nLV (β_time × NOTCH3_ = 0.14, 95% CI 0.034–0.24, *p* = 0.0089) than patients with MR-*NOTCH3* variants (in blue). There was no association between NOTCH3 risk category and 2-year progression of nWMHv (β_time × NOTCH3_ = 0.023, 95% CI −0.015 to 0.060, *p* = 0.22, C) or BPF (β_time × NOTCH3_ = −0.033, 95% CI −0.11 to 0.043, *p* = 0.39, D). BPF = brain parenchymal fraction; HR-NOTCH3 = high-risk *NOTCH3* variant; MR-NOTCH3 = moderate-risk *NOTCH3* variant; MSMD = mean skeletonized mean diffusivity; nLV = normalized lacune volume; nWMHv = normalized white matter hyperintensity volume; β_time_ and *p*_time_ = standardized regression coefficient and *p*-value of the effect of follow-up time; β_time × NOTCH3_ and *p*_time × NOTCH3_ = standardized regression coefficient and *p*-value for the interaction of follow-up time with NOTCH3 risk category after correction for sex, baseline age, and baseline cardiovascular risk factor count (CVRFn).

For the entire cohort, there was an increase in MSMD (β_time_ = 0.20, 95% CI 0.17–0.23, *p* = 7.0 × 10^−24^), nLV (β_time_ = 0.13, 95% CI 0.080–0.19, *p* = 2.1 × 10^−6^), and nWMHv (β_time_ = 0.092, 95% CI 0.075–0.11, *p* = 8.8 × 10^−20^) and a decrease in BPF (β_time_ = −0.22, 95% CI −0.26 to −0.19, *p* = 3.2 × 10^−22^) (Table 2 and Figure 3). Two-year increase in mean MSMD was higher in the HR-NOTCH3 group than in the MR-NOTCH3 group (β_time × NOTCH3_ = 0.074, 95% CI 0.013–0.14, *p* = 0.017), with a 2-year increase in mean MSMD in both the HR-NOTCH3 group (β_time_ = 0.23, 95% CI 0.19–0.27, *p* = 2.5 × 10^−20^) and the MR-NOTCH3 group (β_time_ = 0.16, 95% CI 0.11–0.20, *p* = 5.4 × 10^−10^). MSMD strongly correlated with PSMD, and a sensitivity analysis with PSMD instead of MSMD showed the same results (eFigure 3).

Of 128 participants with HR and MR-*NOTCH3* variants, 42 patients (32.8%) developed at least 1 incident lacune during the follow-up period; this was the case for 33 of 70 patients (47.8%) in the HR-NOTCH3 group and 9 of 58 patients (15.5%) in the MR-NOTCH3 group. Two-year increase in mean nLV was higher in the HR-NOTCH3 group (β_time × NOTCH3_ = 0.14, 95% CI 0.034–0.24, *p* = 0.0089). In fact, nLV did not increase in the MR-NOTCH3 group (β_time_ = 0.056, 95% CI −0.021 to 0.13, *p* = 0.15), and the increase in nLV in the whole cohort was mainly attributable to a strong increase in the HR-NOTCH3 group (β_time_ = 0.19, 95% CI 0.12–0.26, *p* = 1.8 × 10^−7^). There was no association between NOTCH3 risk category and 2-year progression of nWMHv (β_time × NOTCH3_ = 0.023, 95% CI −0.015 to 0.060, *p* = 0.22) or BPF (β_time × NOTCH3_ = −0.033, 95% CI −0.11 to 0.043, *p* = 0.39). Sex and CVRFn did not significantly modify 2-year progression of MSMD, nWMHv, nLV, or BPF.

**Figure 4:**
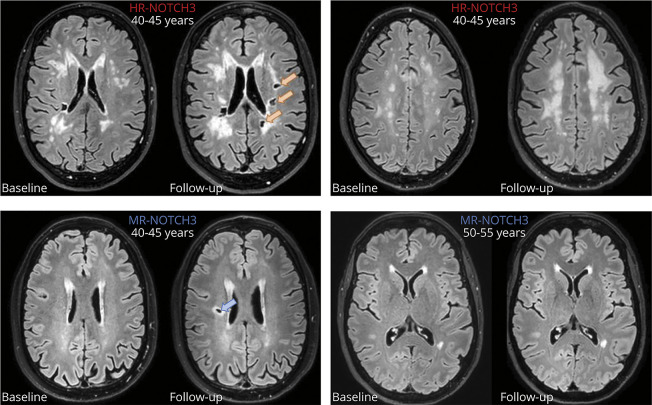
MRI FLAIR Sequences at Baseline and Follow-Up: Examples of Variable Progression of Neuroimaging Markers (A) A patient with an HR-*NOTCH3* variant in their mid 40s developed 12 incident lacunes during the follow-up, of which 3 are shown here (orange arrows); remarkably, this patient did not have any progression of disability on the mRS. (B) A patient with an HR-*NOTCH3* variant in their early 40s whose white matter hyperintensities have increased from patchy to confluent in the semioval center. The patient did not have any incident lacunes, nor did they experience stroke. (C) A patient with an MR-*NOTCH3* variant in their 40s developed 2 lacunes (one of which is indicated with a blue arrow), both in areas with no previous white matter hyperintensities. This patient has a concomitant diagnosis of poorly treated type 1 diabetes. (D) A patient with an MR-*NOTCH3* variant in their early 50s shows a strikingly mild neuroimaging phenotype, with little to no progression with respect to any of the neuroimaging markers. HR-NOTCH3 = high-risk *NOTCH3* variant; MR-NOTCH3 = moderate-risk *NOTCH3* variant, FLAIR: fluid-attenuated inverse recovery; mRS = modified Rankin Scale. To ensure participant anonymity, age ranges are given and sex is not specified.

In a sensitivity analysis, the inference of the effect of NOTCH3 risk category on 2-year progression of MSMD and nLV was not changed when including interactions between follow-up time and age or follow-up time and each CVRF separately or when including pedigree of origin. The effect of NOTCH3 risk category on incident stroke probability and progression of MSMD and nLV remained significant after correction for multiple testing (Table 2). Although the HR-NOTCH3 group progressed significantly faster on several neuroimaging measures than the MR-NOTCH3 group, there was still considerable variability within each NOTCH3 risk category. Illustrative examples of variability in 2-year neuroimaging disease progression for both NOTCH3 risk categories are shown in Figure 4.

### Two-Year Changes in MSMD in Premanifest Participants

Subgroup analysis showed progression of MSMD for individuals younger than 40 years (n = 17, β_time_ = 0.014, 95% CI 0.0093–0.019, *p* = 1.4 × 10^−5^) and for premanifest individuals (n = 24, β_time_ = 0.012, 95% CI 0.0082–0.016, *p* = 1.1 × 10^−6^) (eFigure 4).

## Discussion

In this prospective single-site 2-year follow-up study stratifying for *NOTCH3* variant risk category, we show that NOTCH3 risk category is a major predictor of the rate of clinical and neuroimaging disease progression in patients with CADASIL. Previous longitudinal CADASIL studies did not take NOTCH3 risk category into account.^34-46^ For the whole cohort, clinical and neuroimaging outcome measures significantly progressed in a trial-sensitive time frame of 2 years. Patients with HR-*NOTCH3* variants, however, were shown to progress significantly faster than patients with MR-*NOTCH3* variants with respect to 2-year incident stroke, MSMD, and nLV, after controlling for sex, age, and cardiovascular risk factors at baseline.

Of all outcome measures included in the study, the microstructural marker MSMD showed the most significant 2-year change for the whole cohort, which is in line with a previous study that showed that diffusion MRI had the smallest sample size estimates among clinical and neuroimaging markers in CADASIL.^34^ Diffusion tensor imaging measures have previously been shown to correlate strongly with cognitive outcomes in CADASIL and in SVD in general.^27^ The 2-year increase in MSMD was even significant in young (<40 years) and premanifest individuals. Moreover, there was a significant difference in 2-year MSMD progression between the HR and MR-NOTCH3 groups, with MSMD increasing 46% more in the HR-NOTCH3 group. Differences in progression of neuroimaging markers between the HR and MR-NOTCH3 groups were most pronounced for normalized lacune volume (previously shown to predict clinical worsening in CADASIL^42,44^), which only showed a significant 2-year increase in the HR-NOTCH3 group, with half of the patients having at least 1 incident lacune. We found no significant differences between patients with HR and MR-*NOTCH3* variants for 2-year progression of nWMHv or BPF. There is evidence that WMH may have a heterogeneous etiology in CADASIL, with some hyperintensities being edematous in nature while others are probably ischemic.^47,48^ BPF measures in patients with CADASIL have shown some counterintuitive outcomes, with patients with more severe phenotypes having higher BPF values (suggesting less brain atrophy), which may be explained by the presence of brain swelling masking brain atrophy.^48^

Patients with HR-*NOTCH3* variants had a 4-fold higher incident stroke probability than patients with MR-*NOTCH3* variants, and the lifetime stroke rate was higher in patients with HR-*NOTCH3* variants independent of the number of previous strokes. The 2-year incidence of stroke was 13.4%, which is lower than what has been previously reported in studies with similar follow-up durations (19.8%–22%).^36,43^ This may be attributable to differences in standards of medical care, for example, indication for referral to a neurologist or performing an MRI and, therefore, a stroke diagnosis. A high proportion of patients did have incident lacunes (32.8%), which suggests that, similar to previous studies,^41,44^ covert infarction was frequent in our cohort.

In a multivariable model, only NOTCH3 risk category and age were predictors of 2-year cumulative stroke probability. In line with previously published studies,^36,43,44^ there was a significant 2-year decline of disability and a borderline significant decline in executive function. There was no significant difference in these outcomes between the NOTCH3 risk categories. Likely, measures for disability and cognition are not sensitive enough to capture differences between the groups in such a short time frame. A previous study using a computational approach found a stronger predicted progression on the Matthias Dementia Rating Scale for patients with variants in EGFr domains 7–34 (i.e., predominantly MR and LR-*NOTCH3* variants), compared with patients with EGFr domains 1–6 (i.e., HR-*NOTCH3* variants).^29^ This is in contrast to our results, as we found no single neuroimaging, clinical, or cognitive outcome which progressed faster in the MR-NOTCH3 group than in the HR-NOTCH3 group. This is likely attributable to differences in study design (i.e., a computational vs longitudinal study design) and in NOTCH3 risk category classification (i.e., using EGFr 1–6 vs EGFr 7–34^12^ instead of the updated 3-tiered NOTCH3 risk category classification^13^).

This study has confirmed that, even in a short follow-up period, clinical and neuroimaging disease progression is significantly faster in HR-NOTCH3 patients than in MR-NOTCH3 patients. This has important implications for the development of disease guidelines, supporting a differential approach to HR vs MR-NOTCH3 patients concerning frequency of disease monitoring and disease management, as well as improved individualized disease prediction. Patients with HR-*NOTCH3* variants may benefit from increased clinical surveillance compared with patients with MR-*NOTCH3* variants. Although a patient-centered approach, which, for example, includes age, assessment of cardiovascular risk factors, and disease stage at diagnosis should guide clinical decision making, it is clear that NOTCH3 risk category is an important factor to take into account in individuals genetically diagnosed with a cysteine-altering *NOTCH3* variant.

HR-*NOTCH3* variants have previously been shown to be associated with higher vascular NOTCH3 protein aggregation load.^13,21^ Given the faster rate of short-term disease progression in patients with HR-*NOTCH3* variants, it is tempting to speculate that disease progression is mediated by vascular NOTCH3 aggregation. This potential causal relation between disease progression and increase in vascular NOTCH3 aggregation load merits further study, especially considering the fact that anti-NOTCH3 aggregation approaches for patients with CADASIL are in preclinical development.^49^ Such disease-modifying therapies will likely have the strongest beneficial effect in premanifest individuals, and as vascular NOTCH3 aggregation has been shown to precede clinical symptoms,^50^ NOTCH3 aggregation load could be a promising target-engagement biomarker. Moreover, we show that young and premanifest individuals have a significant 2-year progression in MSMD, which could, therefore, potentially be used as a biomarker in early disease stage intervention clinical trials.

The strength of this study is its prospective, longitudinal nature, with all data uniformly gathered at 1 site, including state-of-the-art neuroimaging acquisition and quantification. A limitation is that we had to make a selection of the most relevant clinical and neuroimaging outcome measures to decrease multiple testing effects. We selected neuroimaging markers which have been shown to be consistently present in patients with CADASIL or are strongly correlated with disease outcome measures.^26,27,42,45^ The effect of NOTCH3 risk category on progression of other neuroimaging markers, for example, CMB or PVS, merits further research. Likewise, to limit the number of independent variables in the prediction models, we did not study the effect of individual CVRF on disease progression, but instead used the total burden of CVFR as a predictor. Finally, given the limited number of patients with LR-*NOTCH3* variants in our cohort, which is because low-risk variants are rare in CADASIL pedigrees, we could not determine disease progression in this subgroup of patients.

In conclusion, we show that patients with CADASIL show a significant progression of neuroimaging markers, executive function, and disability in a clinical trial-sensitive time frame of 2 years. We show the importance of taking *NOTCH3* variant risk category into account because patients with HR-*NOTCH3* variants have a significantly faster disease progression compared with patients with MR-*NOTCH3* variants. This has important implications for disease prediction and monitoring in the clinic, biomarker development, and selection of patients and outcome measures in clinical trials.

## Appendix Authors

**Table:**

Name	Location	Contribution
Minne N. Cerfontaine, MD	Department of Clinical Genetics, Leiden University Medical Center, the Netherlands	Drafting/revision of the manuscript for content, including medical writing for content; major role in the acquisition of data; study concept or design; analysis or interpretation of data
Remco J. Hack, MD	Department of Clinical Genetics, Leiden University Medical Center, the Netherlands	Drafting/revision of the manuscript for content, including medical writing for content; major role in the acquisition of data; analysis or interpretation of data
Benno Gesierich, PhD	Medical Image Analysis Center (MIAC) and Department of Biomedical Engineering, University of Basel, Switzerland	Drafting/revision of the manuscript for content, including medical writing for content; analysis or interpretation of data
Marco Duering, MD	Medical Image Analysis Center (MIAC) and Department of Biomedical Engineering, University of Basel, Switzerland	Drafting/revision of the manuscript for content, including medical writing for content; analysis or interpretation of data
Marie-Noëlle W. Witjes-Ané, PhD	Department of Geriatrics and Psychiatrics, Leiden University Medical Center, the Netherlands	Drafting/revision of the manuscript for content, including medical writing for content
Mar Rodríguez-Girondo, PhD	Department of Medical Statistics, Leiden University Medical Center, the Netherlands	Analysis or interpretation of data; study concept or design
Gido Gravesteijn, MD, PhD	Department of Clinical Genetics, Leiden University Medical Center, the Netherlands	Drafting/revision of the manuscript for content, including medical writing for content; study concept or design; analysis or interpretation of data
Julie Rutten, MD, PhD	Department of Clinical Genetics, Leiden University Medical Center, the Netherlands	Drafting/revision of the manuscript for content, including medical writing for content; major role in the acquisition of data; study concept or design; analysis or interpretation of data
Saskia A.J. Lesnik Oberstein, MD, PhD	Department of Clinical Genetics, Leiden University Medical Center, the Netherlands	Drafting/revision of the manuscript for content, including medical writing for content; major role in the acquisition of data; study concept or design; analysis or interpretation of data

## Glossary

BPF: brain parenchymal fraction
CADASIL: cerebral autosomal dominant arteriopathy with subcortical infarcts and leukoencephalopathy
CLMM: cumulative link mixed model
CVRF: cardiovascular risk factors
CVRFn: number of cardiovascular risk factors at baseline
DiViNAS: Disease Variability in *NOTCH3*-Associated Small Vessel Disease
DTI: diffusion tensor imaging
EGFr: epidermal growth factor like repeat
FLAIR: fluid-attenuated inversion recovery
HR/MR/LR-NOTCH3: high risk/moderate risk/low risk *NOTCH3* variants
IQR: interquartile range
LMM: linear mixed model
mRS: modified Rankin Scale
MSMD: mean skeletonized mean diffusivity
OR: odds ratio
nLV: normalized lacune volume
nWMHv: normalized white matter hyperintensity volume
PSMD: peak width of the skeletonized mean diffusivity
SVD: small vessel disease
STROBE: Strengthening the Reporting of Observational studies in Epidemiology
SWI: susceptibility-weighted image
TMT: Trail Making Test
TMT_B/A_: Trail Making Test B given A *t*-scores
WMH: white matter hyperintensity
